# Presentation, Etiology, Outcome, and Differentiation of Visual Semiology of Adult Occipital Epilepsy From Visual Aura of Migraine Headache: A Prospective Study in a Tertiary Care Center in Bangladesh

**DOI:** 10.7759/cureus.24186

**Published:** 2022-04-16

**Authors:** Reaz Mahmud, Hashmi Sina

**Affiliations:** 1 Neurology, Dhaka Medical College Hospital, Dhaka, BGD

**Keywords:** occipital lobe, aura, migraine, headache, epilepsy

## Abstract

Background: Occipital epilepsy is an uncommon and likely underdiagnosed type of epilepsy that is often misdiagnosed as a migraine with aura. High clinical suspicion and subsequent electroencephalogram (EEG) and brain imaging lead to early diagnosis.

Methods: We recruited patients with occipital epilepsy based on visual semiology, structural abnormalities in the occipital region, or EEG changes who visited the Department of Neurology of Dhaka Medical College from June 2019 to January 2020. We documented the presentations, etiology, and outcomes at the 12-month follow-up. Additionally, we compared the clinical features of patients with occipital epilepsy (n = 10) and those with migraine with aura (n = 18).

Results: We identified three and seven cases of idiopathic and symptomatic occipital epilepsy, respectively, all presenting with visual semiology. Symptomatic occipital epilepsy occurred due to space-occupying lesions, post-hypoxic damage, post-stroke encephalomalacia, gyral calcification from Sturge-Weber syndrome, and Wilson’s disease. Age, sex, illness duration, headache severity, and associated features were similar between the migraine with aura and epilepsy groups. In occipital epilepsy, the median (IQR) age was 22 (15-47) years, and the patients were predominantly female (8, 80%). The visual auras lasted 35 (3-375) seconds and included colored dots or light flashes that persisted for seconds (50%) before (60%), during (30%), and after (10%) the headache. Compared to migraines, the headaches were global (90%), compressive (90%), and of shorter duration (210 minutes, IQR: 150-630). Except for nausea or osmophobia, vomiting (80%), photophobia (80%), and phonophobia (70%) occurred. Most cases had associated focal or bilateral tonic-clonic seizures (60%; p-value < 0.001). In contrast, the visual auras in migraine were scotomas, white or golden dots, or light flashes lasting for minutes (83.3%; p-value = 0.02) before the headache. The headaches lasted longer (720 minutes, IQR: 345-1,440, p-value < 0.03), were unilateral (44%) or bilateral (50%), and throbbing (72%; p-value = 0.003). Headache was associated with photophobia (94.4%; p-value = 0.28), phonophobia (88.9%; p-value 0.31), and osmophobia (38.9%; p-value 0.03); no associated convulsions occurred. At the 12-month follow-up, most occipital epilepsy patients (9, 90%) responded well to carbamazepine.

Conclusions: Patients with transient and distinct elementary visual hallucinations headache characteristics different from migraines with associated convulsions warrant evaluation for occipital epilepsy.

## Introduction

Occipital lobe epileptic foci give rise to occipital seizures that manifest with visual semiology [1]. Occipital epilepsy is an uncommon and underdiagnosed type of epilepsy that affects 1.2-2.5% of all newly diagnosed epileptic patients [2] and is often misdiagnosed as migraine with aura [3]. In adults, occipital epilepsy is more uncommon, accounting for only 1% of all cases of epilepsy [4]. Idiopathic occipital epilepsy mainly manifests in children as idiopathic early-onset childhood epilepsy with occipital spikes, late-onset childhood epilepsy with occipital spikes, and idiopathic photosensitive epilepsy [2]. In contrast, few studies of occipital epilepsy in adults exist.

Auras in migraine are mostly visual (90%) [5]. The underlying pathophysiology of migraine headache is a cortical spreading phenomenon that results from the efflux of cellular potassium and glutamate [6]. Migraine is the third leading cause of morbidity for both sexes under 50 years of age and affects approximately 12% of the population [7]. The International Headache Society has described migraine aura-triggered seizures as those in which the aura triggers a seizure episode [5].

Auras are also present in focal onset impaired awareness seizures (previously known as complex partial seizures) [8]. Focal epileptiform discharges confined to the occipital cortex may cause occipital seizures and spread to the adjacent cortex, resulting in a focal onset impaired awareness seizure or focal to bilateral seizures [1]. In occipital seizures, migraine-like headaches and ictal vomiting occur within 3-15 min after focal seizures [2,3].

Visual phenomena, convulsions, headaches, and vomiting occur in both migraines with aura and occipital lobe epilepsy. Thus, the most important differential diagnosis of occipital epilepsy is migraine. However, differentiating between them is difficult without meticulous history-taking.

In cases of headache consistent with migraine with aura, neuroimaging is unnecessary [9]. Conversely, occipital epilepsy syndrome may be idiopathic, cryptogenic, or symptomatic; 30% of cases are symptomatic [3]. In these cases, neuroimaging is mandatory to determine the etiology. Differentiating the two diseases based on patient history and presentation will help rationalize the choice of investigation.

Furthermore, treatment modalities often differ in these diseases. Although carbamazepine is the treatment of choice for occipital epilepsy [1], it is less supported or ineffective in migraines [10]. Prophylactic drugs for migraine include topiramate, sodium valproate, and amitriptyline. Therefore, a missed diagnosis of occipital epilepsy will lead to unnecessary treatment and long-term suffering.

Here, we described the presentations, etiology, and 12-month treatment outcomes of occipital epilepsy in adults. Additionally, we highlighted findings to differentiate visual auras in migraine from the occipital seizure semiology of occipital epilepsy.

## Materials and methods

This was a prospective cohort study conducted in the headache and epilepsy clinics of the Neurology Department of Dhaka Medical College from June 1, 2019 to November 30, 2021. Ethical approval was obtained from the ethical review committee of Dhaka Medical College (ERC-DMC/ECC/2021/269). Informed written consent was obtained from all study participants. We included most of the patient's data in the manuscript; the rest will be made available on reasonable request from the corresponding author.

We documented patients with occipital epilepsy who presented with suggestive visual semiology and/or electroencephalogram (EEG) changes and structural abnormalities in the occipital region and visited the Department of Neurology, Dhaka Medical College, between June 2019 and January 2020. In all cases where there were no confirmed EEG changes, such instances suggested visual semiology and structural changes in the EEG helped in diagnosis. We followed up with the patients for at least 12 months to determine their outcomes. Additionally, we compared ictal visual hallucinations to auras in 18 patients with migraines.

Participants and clinical and laboratory evaluations

All patients presented with ictal visual hallucinations, headaches, and vomiting. We diagnosed occipital epilepsy based on the characteristics of the ictal phenomenon, imaging abnormalities in the occipital region, or epileptiform discharges in the occipital region on EEG. We had the prospective cohort (followed up for at least one year) of occipital epilepsy only. We randomly selected 18 patients with migraines with visual aura who agreed to participate in the study to identify the differences in presentation and visual semiology. These patients had undergone at least one brain magnetic resonance imaging (MRI) assessment and EEG during sleep and awake states conducted by the radiology department and electrophysiology lab of Dhaka Medical College, respectively. We recorded the EEG according to the international 10-20 system with 21 electrodes. The sensitivity was 7 μV/mm, the low-frequency filter was set at 1 Hz, the high-frequency filter at 70 Hz, and the digital display was 10 seconds/page. The waking record contained at least 20 minutes of artifact-free recording at rest, including a brief period when eyes are open. The period did not include the time required for hyperventilation, photic stimulation, and sleep recording. The EEG was recorded during and at least one minute after hyperventilation. Flashes in photic stimulation were given at varying rates, including 1, 3, 5, 10, 13, 15, 17, 20, and 25 Hz in trains lasting 10 seconds. We tried to obtain at least 20 minutes of sleep recording but were not always successful. The total recording time was 45-50 minutes. We did not perform a-priori sample size calculations as we sought to include as many patients as possible. Post hoc analysis was done using the formula n = Z2pq/d2, where z = 1.96 (at 95% confidence level), p = 2%, and d = allowable error/precision = 10%. The estimated prevalence was considered to be 2%, which was based on population studies that have indicated that 1.2-2.6% of epilepsy cases are occipital epilepsy [2]. It revealed that a sample size of 10 patients can detect changes with 90% precision and a 95% confidence interval in two-tailed tests. We did not make any power calculations. We then compared the ictal visual phenomena to migraine auras.

Study design and procedure

We examined all patients through interviews to complete a case record form. We documented details about the visual phenomena, including their character, color, and duration. We also recorded the characteristics of the headache, associated features, the phenomenology of convulsions (if present), precipitating factors, and a previous history of illnesses that may involve the occipital lobe. We defined migraine headache, visual aura of migraine, and migraine-triggered seizure using the International Classification of Headache Disorders, 3rd edition [5]. The definition of seizures and the terminology used in this study were based on the operational classification of seizure types from the International League Against Epilepsy [11]. We defined occipital epilepsy as epilepsy presenting with suggested visual semiology, structural abnormalities in the occipital region, and EEG changes. Regarding the duration of events, the responder gave the range of duration. For statistical analysis, we considered the highest value in the range.

Statistical analysis

We analyzed the data using IBM SPSS Statistics for Windows, version 20.0 (IBM Corp., Armonk, NY). We expressed qualitative data as numbers and percentages, quantitative data with normal distribution as means (standard deviation), and non-normal data as medians (interquartile range, IQR). We compared occipital epilepsy and migraine with aura using Fisher's exact or Chi-square tests (as appropriate) for qualitative data, unpaired t-tests for normally distributed quantitative data, and the Mann-Whitney U tests for non-normal quantitative data. Statistical significance was set at p < 0.05.

## Results

**Figure 1:**
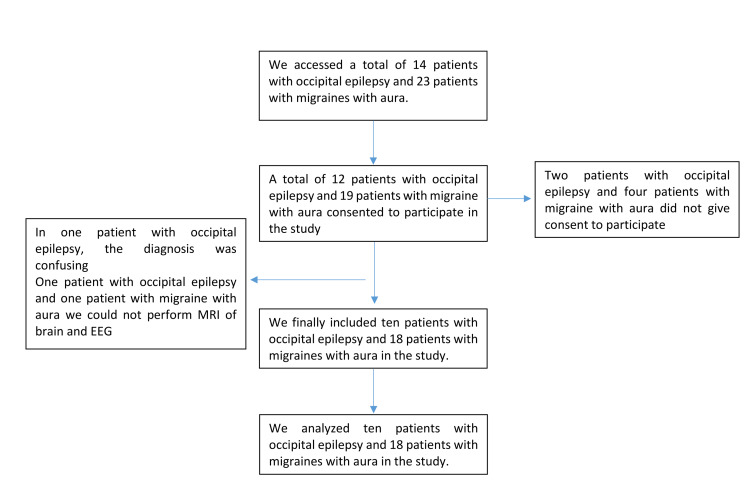
Patient selection for this study

We assessed 14 and 23 patients with occipital epilepsy and migraine with aura, respectively. After excluding those who did not provide consent, those who had a confusing diagnosis, and those who did not undergo brain MRI and EEG, 10 and 18 patients with occipital epilepsy and with migraine with aura, respectively, were included in the analysis (Figure 1).

The median age of the patients with occipital epilepsy (22 years, IQR: 15-56) and migraine with aura (30 years, IQR: 23.5-36.25) were similar (p = 0.56). In both patient groups, women were predominantly affected (occipital epilepsy, 8 [80%]; migraine with aura, 15 [83.3%]; p > 0.99).

The median symptom duration was similar in the occipital epilepsy (30 months, IQR: 19.5-99) and migraine with aura (30 months, IQR: 22-72) groups (p = −0.87). The median duration of the visual phenomenon of occipital epilepsy was shorter (35 seconds, IQR: 3-375) than that of migraine with aura (460 seconds, IQR: 225-1,800), and the difference was significantly different (p = 0.03). In most patients with occipital epilepsy (n = 5, 50%), the visual phenomenon persisted for seconds compared to minutes in those with migraine with aura (n = 15, 83.3%; p = 0.02).

All patients with migraine with aura (17, 94.4%) reported that the phenomenon occurred before the headache. In contrast, patients with occipital epilepsy reported that the phenomenon occurred concurrently (n = 3, 30%) or after (n = 1, 10%) the headache. Other associated symptoms included vertigo, tinnitus, dysarthria, diplopia, hemiparesthesia, and hemiparesis; the cumulative frequency of these symptoms was similar between occipital epilepsy and migraine with aura groups. Throbbing headache was most common in the migraine with aura group (n = 13, 72.2%) compared to compressive headache in the occipital epilepsy group (n = 9, 90%; p = 0.003). The incidence of global headache was higher in the occipital epilepsy group (n=9, 90%) than in the migraine with aura group (n = 9, 50%; p = 0.10). Headache severity, which was assessed using a visual analog score that went up to 10, was also similar in both groups. The median (IQR) headache severity scores for occipital epilepsy and migraine with aura groups were 7.5 (6.7-8) and 8 (7-8), respectively (p = 0.38).

The headache persisted for a longer duration in the migraine with aura group (720 minutes, IQR: 345-1,440) than in the occipital epilepsy group (210 minutes, IQR: 150-630; p = 0.03). Patients in both groups experienced photophobia and phonophobia with the headache. Osmophobia (n = 7, 38.9%) occurred only in patients with migraine with aura (p = 0.03). Vomiting with headache occurred in patients in both groups; however, nausea occurred less frequently in the occipital epilepsy group (6 [60%] vs. 17 [94.4%], p = 0.04). Focal to bilateral seizure episodes occurred exclusively in the occipital epilepsy group (n = 6, 60%; p = 0.001; Table 1).

**Table 1:** Comparisons of demographic and clinical characteristics between patients with occipital epilepsy and those with migraine with aura ^a^Mann-Whitney U test. ^b^Epilepsy presented with visual semiology and, or structural abnormality in the occipital region, EEG changes. ^c^According to the International Classification of Headache Disorders, 3rd edition. ^d^The sensation of rotation in space or of surrounding objects. ^e^The sensation of hearing noises in the ear is audible only to the patients. ^f^VAS, visual analogue scale 1-10. ^g^Chi-square test. ^h^Fisher's exact test. ^i^Seconds more than one second to less than a minute, minutes-more than one minute to less than an hour, hours-more than one hour.

Characteristics	Occipital epilepsy^b^ (N = 10)	Migraine with aura^c^ (N = 18)	P-value
Age, median (IQR)	22 (15–56)	30 (23.5–36.25)	0.56^a^
Sex, n (%)	8 (80)	15 (83.3)	>0.99
Illness duration in months, median (IQR)	30 (19.5–99)	30 (22­–72)	0.87^a^
Duration of the visual phenomenon in seconds, median (IQR)	35 (3–375)	460 (225–1800)	0.04^a^
Relationship of visual phenomenon with headache			
Visual phenomenon before a headache, n (%)	6 (60)	17 (94.4)	
Visual phenomenon and headaches simultaneous, n (%)	3 (30)	0 (0)	0.03^g^
Visual phenomenon after a headache, n (%)	1 (10)	0 (0)	
Number of symptoms other than visual phenomenon			
One	1 (10)	6 (33.3)	
Two	4 (40)	6 (33.3)	0.57^g^
Three	4 (40)	4 (22.2)	
Four	0 (0)	1 (5.6)	
Five	1 (10)	19 (5.6)	
Duration of visual phenomenon^i^			
Seconds	5 (50)	1 (5.6)	
Minutes	4 (40)	15 (83.3)	0.02^g^
Hours	1 (10)	2 (11.1)	
Headache type			
Throbbing, n (%)	1 (10)	13 (72.2)	0.003^h^
Compressive, n (%)	9 (90)	4 (22.2)	
Site of headache			
Unilateral, n (%)	1 (10)	8 (44.4)	0.10^h^
Global, n (%)	9 (90)	9 (50)	
Duration of headache in minutes, median (IQR)	210 (150–630)	720 (345–1,440)	0.03^a^
VAS severity^f^	7.5 (6.7–8)	8 (7–8)	0.38^a^
Associated symptoms other than visual phenomenon			
Vertigo^d^, n (%)	6 (60)	10 (55.6)	>0.99^h^
Tinnitus^e^, n (%)	5 (50)	1 (5.6)	0.013^h^
Dysarthria, n (%)	1 (10)	5 (27.8)	0.37^h^
Diplopia, n (%)	2 (20)	4 (22.2)	>0.99^h^
Hemiparaesthesia, n (%)	2 (20)	4 (22.2)	>0.99^h^
Hemiparesis, n (%)	2 (20)	1 (5.6)	0.28^h^
Associated phenomenon			
Photophobia, n (%)	8 (80)	17 (94.4)	0.28^h^
Phonophobia, n (%)	7 (70)	16 (88.9)	0.31^h^
Osmophobia, n (%)	0	7 (38.9)	0.03^h^
Nausea, n (%)	6 (60)	17 (94.4)	0.04^h^
Vomiting, n (%)	8 (80)	8 (44.4)	0.11^h^
Generalized convulsion, n (%)	6 (60)	0	0.001^h^

The visual phenomena in patients with occipital epilepsy included flashes of bright red light (cases 1, 2, 5, and 7), multi-colored light (case 3), small, bright, and multi-colored dots (case 4), and bright flickering light (case 10). Two patients experienced hallucinations of objects such as a palm (case 9) and a frightening human image (case 6) (Table 2).

**Table 2:** Clinical data of patients with occipital epilepsies and migraine with aura M: male, F: female, EEG: electroencephalogram; MRI: magnetic resonance imaging; FLAIR: fluid-attenuated inversion recovery

No	Age (Years)	Sex	Description of the visual phenomenon	Features other than visual phenomenon	Imaging	EEG findings	Type of occipital epilepsy	Associated disease	Treatment and follow-up
1	14	M	Flashes of bright red light in the right eye followed by transient visual loss; duration: 3–5 seconds	Vertigo, post-ictal severe compressive headache followed by aura associated with photophobia, no generalized convulsion	Cortical dysplasia in the left parieto-occipital region (Figure 2E)	Generalized epileptiform discharges (Figure 3A)	Symptomatic occipital epilepsy	None	Carbamazepine; Seizure-free after 6 months
2	27	M	Flashes of bright red light in the left eye simultaneously with headache; duration: 1–2 seconds	Concomitant severe compressive global headache with aura associated with photophobia, phonophobia, diplopia, and vomiting. Associated focal convulsion in the right hand and face. Occasional transient unconsciousness at the height of headache severity	Arachnoid cyst in the right hippocampal area (Figure 2F)	Left-sided focal occipital discharge (Figure 3 B)	Idiopathic occipital epilepsy		Carbamazepine; seizure-free after 1 month
3	17	F	Flashes of multi-colored light in the right eye, sometimes colored arcs; duration: 1–3 seconds	Vertigo, tinnitus, concomitant severe compressive global headache with aura associated with nausea, vomiting, photophobia, phonophobia	Normal MRI	Epileptiform discharges from both occipital regions (Figure 4A)	Idiopathic occipital epilepsy		Treated with sodium valproate. Frequency reduced to two attacks per month after 6 months of follow-up.
4	61	F	Small, bright multi-colored dots in both eyes; duration: 5–10 seconds	Vertigo, tinnitus, severe unilateral throbbing headache associated with nausea, vomiting, photophobia, phonophobia	FLAIR and T2 hyperintensity in both occipital regions (Figure 2C-2D)	Epileptiform discharges from both occipital regions	Symptomatic occipital epilepsy	Eclampsia at 25 years of age	Carbamazepine; Seizure-free within 2 months
5	15	F	Flashes of bright light in both eyes, followed by transient visual loss; duration: 1–3 seconds	Vertigo, tinnitus, diplopia, hemiparesis, severe compressive global headache associated with nausea, vomiting, photophobia, phonophobia, and generalized convulsions	Multiple ring-enhancing lesions in the right occipital region (Figure 2A-2B)	Generalized epileptiform discharge (Figure 4B)	Symptomatic occipital epilepsy		Referred to neurosurgery and lost to follow-up
6	45	F	Formed visual hallucination of an abnormal and frightening human image; duration: 50–60 seconds	Severe compressive global headache after the aura associated with disruption of visual images and generalized convulsions	Normal brain MRI	Generalized epileptiform discharges	Idiopathic occipital epilepsy		
7	60	F	Flashes of multi-colored light in the right eye; duration: 500–600 seconds	Vertigo, tonic deviation of eyes to right with turning of the head, severe compressive global headache associated with nausea, vomiting, photophobia, phonophobia	Encephalomalacia in the left occipital region (Figure 2G)	Normal EEG	Symptomatic occipital epilepsy	Ischemic stroke with right hemianopia	Seizures were uncontrolled despite increasing the dose of carbamazepine
8	17	F	Multi-colored light in both eyes with visual blurring; duration: 200–300 seconds	Tinnitus, paresthesia, palilalia at seizure onset. Severe compressive global headache associated with generalized convulsions.	Bilateral sclerosis in the occipital region (Figure 2J)	Right focal epileptiform discharges and slow wave with generalized epileptiform discharge (Figure 5A)	Symptomatic occipital epilepsy	Convulsion due to meningitis at 1.5 months of age with hypoxic encephalopathy	The seizure frequency was reduced to 1–2 seizures after 12 months of treatment with escalating dosage of carbamazepine
9	15	F	Objects such as a plum; duration: 150–180 seconds	Tinnitus, paresthesia, severe compressive global headache, associated with nausea, vomiting, photophobia, phonophobia, and generalized convulsions	T2 and FLAIR hyperintensity in both the basal ganglia and right occipital cortex (Figure 2K-2L)	Bi-frontal slow wave with right-sided occipital epileptiform discharges.	Symptomatic occipital epilepsy	Wilson’s disease	Died after one month due to hepatic encephalopathy; levetiracetam was given
10	55	F	Flickering bright light in the left eye; duration: 300–360 minutes	Vertigo, severe compressive global headache, associated with, nausea, vomiting, photophobia, phonophobia, focal left hand convulsion	Gyral calcification in the left occipital region (Figure 2H-2I)	Loss of normal background rhythm in the left occipital cortex. Right-sided occipital slow wave (Figure 5B).	Symptomatic occipital epilepsy	Sturge–Weber syndrome	Carbamazepine; she became seizure-free after 5 months
11	36	M	The patient experienced scintillating scotoma in both eyes; duration: 240–300 seconds	No headache	Normal MRI of brain	Normal EEG	Migraine aura sine headache	Had a history of left acoustic neuroma (operated)	
12	25	F	The patient experienced flashing bright white lights with visual blurring; duration: 4–5 seconds	Severe throbbing unilateral headache associated with nausea, vomiting, photophobia, phonophobia, osmophobia	Normal MRI of brain		Migraine with aura		
13	26	F	The patient experienced an arch in front of the right eye with the occasional feeling of uneven objects; duration: 200–300 seconds	Right-sided hemianopia, severe unilateral throbbing headache associated with nausea, vomiting, photophobia, phonophobia, osmophobia	Normal MRI of brain	Normal EEG	Migraine with aura		
14	40	F	The patient experienced flickering white lights in both eyes; duration: 1,500–1,800 seconds	Dizziness, transient monocular blindness, severe compressive global headache, associated with nausea, vomiting photophobia, phonophobia, osmophobia	Normal MRI of brain	Normal EEG	Migraine with aura (retinal migraine)		
15	20	F	The patient experienced small white bright dots in both eyes; duration: 500–600 seconds	Severe throbbing global headache associated with nausea, vomiting, photophobia, phonophobia	Normal MRI of brain	Normal EEG	Migraine with aura		
16	18	F	The patient experienced flashes of bright white light in both eyes; duration: 1,500–1,800 seconds	Vertigo, severe throbbing global headache associated with nausea, photophobia, phonophobia	Normal MRI of brain	Normal EEG	Migraine with aura		
17	37	F	The patient experienced flashes of bright light with white dots; duration: 240–300 seconds	Vertigo, diplopia, hemi paresthesia, severe compressive global headache associated with nausea, photophobia, phonophobia	Normal MRI of brain	Normal EEG	Migraine with aura (migraine with brainstem aura)		
18	32	F	The patient experienced small bright golden dots in both eyes; duration: 100–120 seconds	Vertigo, severe throbbing global headache associated with nausea, vomiting, photophobia, phonophobia	Normal MRI of brain	Normal EEG	Migraine with aura		
19	30	F	The patient experienced small bright dots in both eyes; duration: 500–600 seconds	Vertigo, dysarthria, transient loss of consciousness, severe throbbing unilateral headache associated with nausea, photophobia, phonophobia, osmophobia	Normal MRI of brain	Normal EEG	Migraine with aura (migraine with brainstem aura)		
20	30	F	The patient experienced small bright dots and occasional scintillating scotoma in both eyes; duration: 500–600 seconds	Vertigo, dysarthria, diplopia, severe throbbing unilateral headache associated with nausea, photophobia, phonophobia	Normal MRI of brain	Normal EEG	Migraine with aura (migraine with brainstem aura)		
21	30	F	The patient experienced small bright white dots in the right eye; duration: 500–600 seconds	Vertigo, dysarthria, hemiparesthesia, severe throbbing unilateral headache associated with nausea, phonophobia, osmophobia	Normal MRI of brain	Normal EEG	Migraine with aura (migraine with brainstem aura)	insomnia	
22	24	F	The patient experienced flashes of bright white light with dots; duration: 300–320 seconds	Vertigo, tinnitus, severe compressive global headache associated with nausea photophobia, phonophobia	Normal MRI of brain	Normal EEG	Migraine with aura (migraine with brainstem aura)		
23	27	F	No visual aura	Vertigo, palpitations, facial deviation to right, severe compressive global headache associated with nausea, vomiting, photophobia, phonophobia	Normal MRI of brain	Normal EEG	Sporadic hemiplegic migraine (SHM)		
24	22	F	The patient experienced flashes of golden-colored lights in both eyes; duration: 50–60 seconds	Diplopia, paresthesia, transient loss of consciousness, severe throbbing global headache associated with nausea, photophobia, phonophobia, osmophobia	Normal MRI of brain	Normal EEG	Migraine with aura (migraine with brainstem aura)		
25	20	F	The patient experienced flashes of white light with visual blurring in both eyes; duration: 1800-1900 seconds	Vertigo, dysarthria, diplopia, hemi paresthesia, hemiparesis, severe throbbing global headache associated with nausea, vomiting, photophobia, phonophobia, osmophobia	Normal MRI of brain	Normal EEG	Sporadic hemiplegic migraine (SHM)		
26	40	F	The patient experienced flashes of bright white light in the left eye; duration: 250-300 seconds	The unilateral severe throbbing headache followed by aura was associated with nausea, vomiting, photophobia, phonophobia	Normal MRI of brain	Normal EEG	Migraine with aura		Hypertension
27	35	M	The patient experienced flashes of bright white light with the central scotoma in the right eye; duration: 3000-3600 seconds	Dysarthria, Vertigo, the severe unilateral throbbing headache followed by aura associated with, nausea, photophobia, phonophobia	Normal MRI of brain	Normal EEG	Migraine with aura (migraine with brainstem aura)		
28	60	M	The patient experienced flashes of bright light in both eyes; duration: 3000-3600 seconds	The severe throbbing unilateral headache followed by aura associated with monocular blindness, nausea, photophobia	Normal MRI of brain	Normal EEG	Migraine with aura (retinal migraine)		

**Figure 2:**
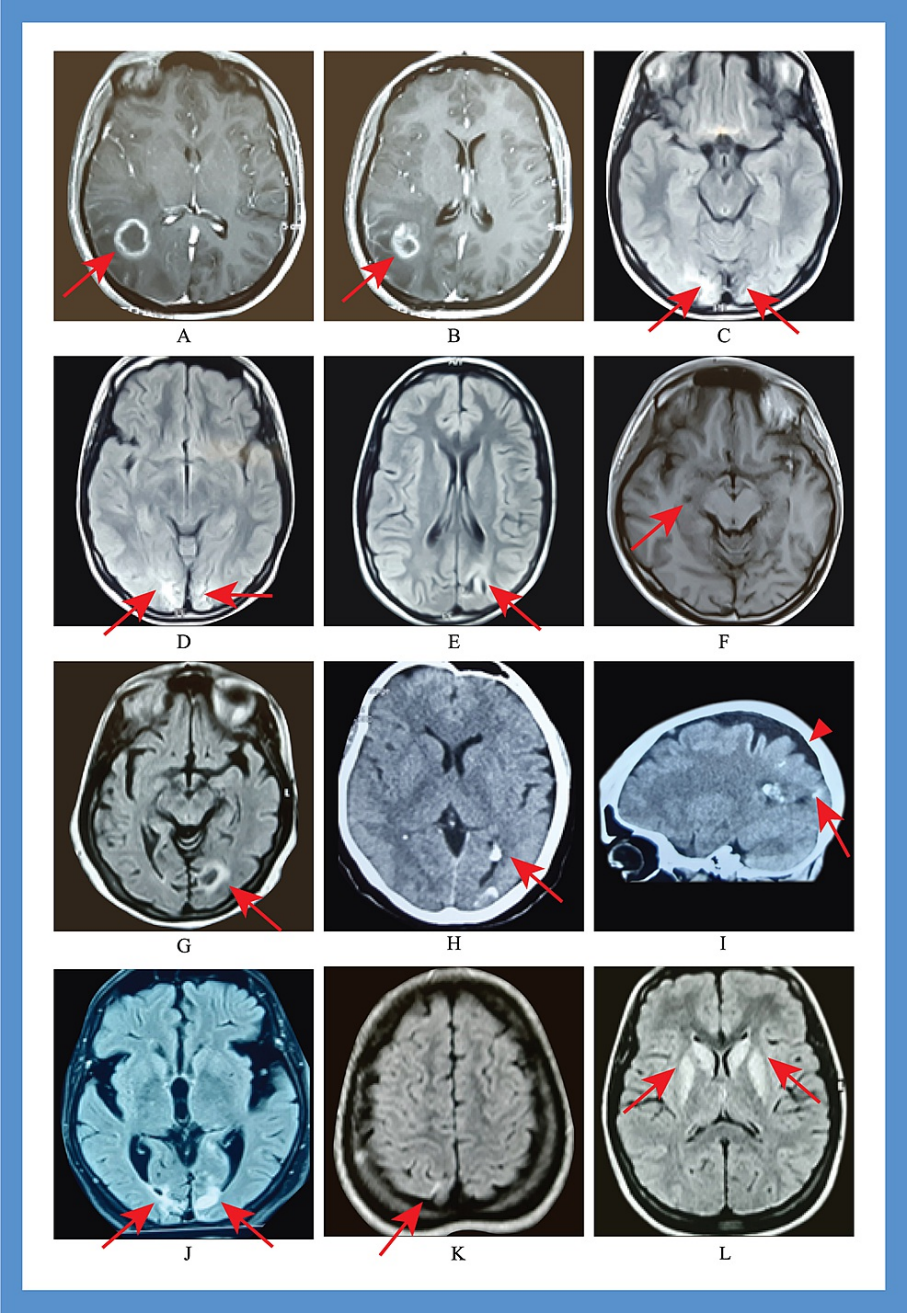
Brain imaging of the different patients with occipital epilepsy (A, B) MRI of the brain with contrast showing multiple rings enhancing lesions in the right parieto-occipital region (case 5; red arrow); (C, D) MRI of the brain showing sclerosis in both occipital poles marked on the right side (case 8; red arrow; (E) MRI of the brain showing focal cortical dysplasia in the left occipital region (case 1; red arrow); (F) MRI of the brain showing arachnoid cyst in the right hippocampal region (case 2; red arrow); (G) MRI of the brain with encephalomalacia and marginal sclerosis in the left occipital region (case 7; red arrow); (H, I) CT scan of the brain showing gyral calcification in the left occipital region (case 10; red arrow) and associated cortical atrophy (arrowhead); (J) MRI of the brain showing sclerosis in the occipital region (case 8; red arrow); (K, L) MRI of the brain showing FLAIR hyperintensities in the caudate nucleus, putamen, and left occipital region (J) (case-9; red arrow).

**Figure 3:**
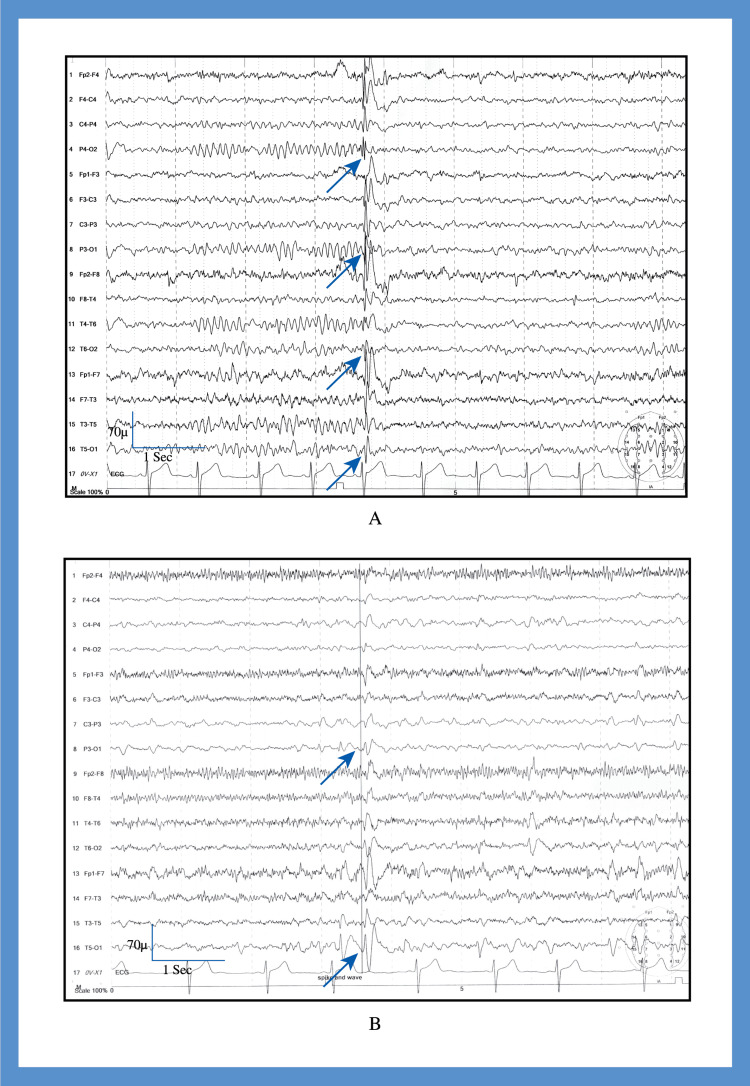
Electroencephalogram of patients with occipital epilepsy - cases 1 and 2 (A) Case 1, recorded in sleep with sensitivity 70 µV. The Longitudinal bipolar montage revealed generalized epileptiform discharges (arrow). (B) Case 2, recorded in sleep with sensitivity 70 µV. The longitudinal bipolar montage revealed focal epileptiform discharges from the left occipital region (arrow).

**Figure 4:**
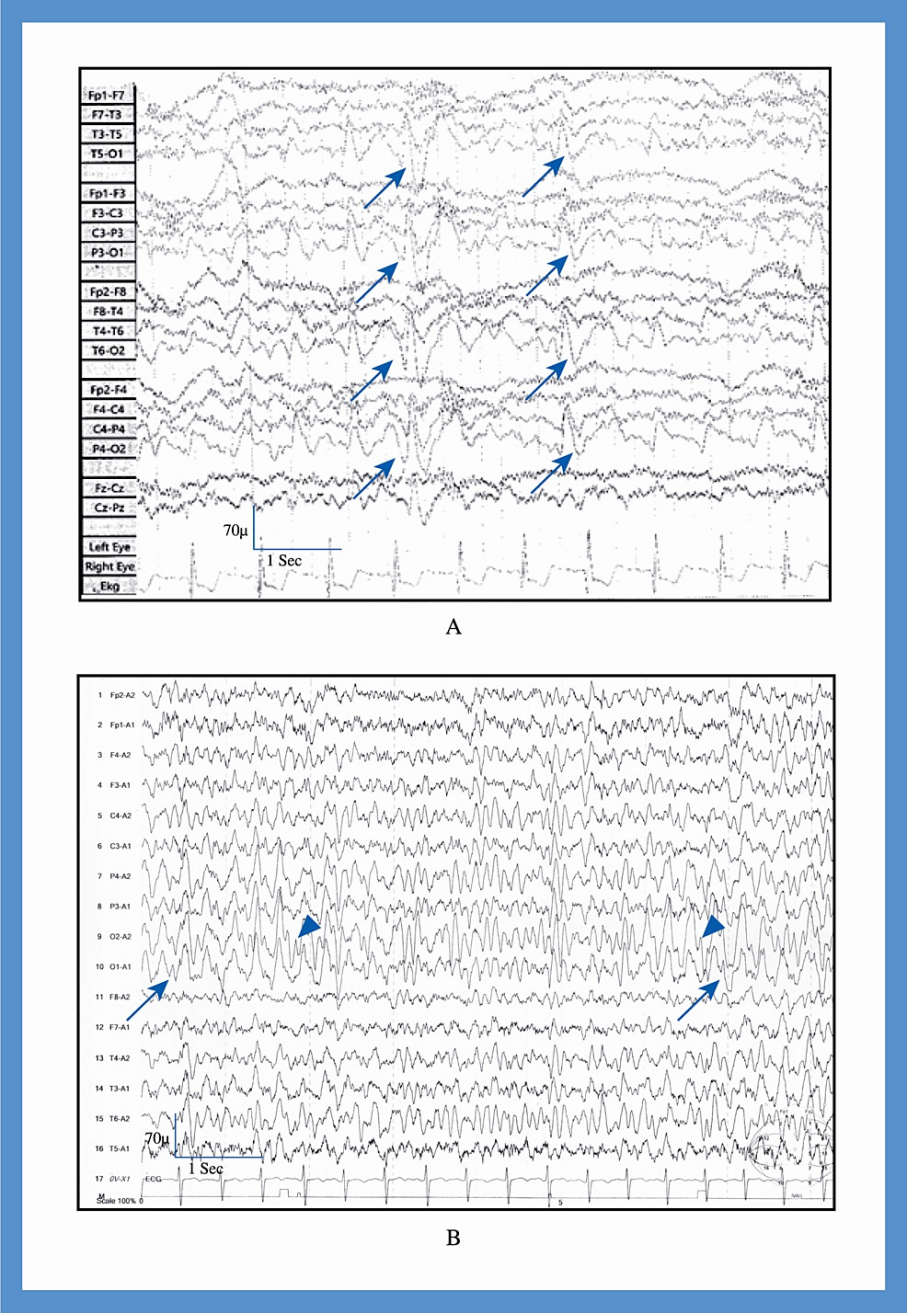
Electroencephalogram of patients with occipital epilepsy - cases 3 and 4 (A) Case 3, recorded in sleep with sensitivity 70 µV. The longitudinal bipolar montage revealed bilateral occipital epileptiform discharges (arrow). (B) Case 4, recorded in awake with sensitivity 70 µV. The referential montage revealed bilateral occipital epileptiform discharges (arrow-left, arrow head-right).

**Figure 5:**
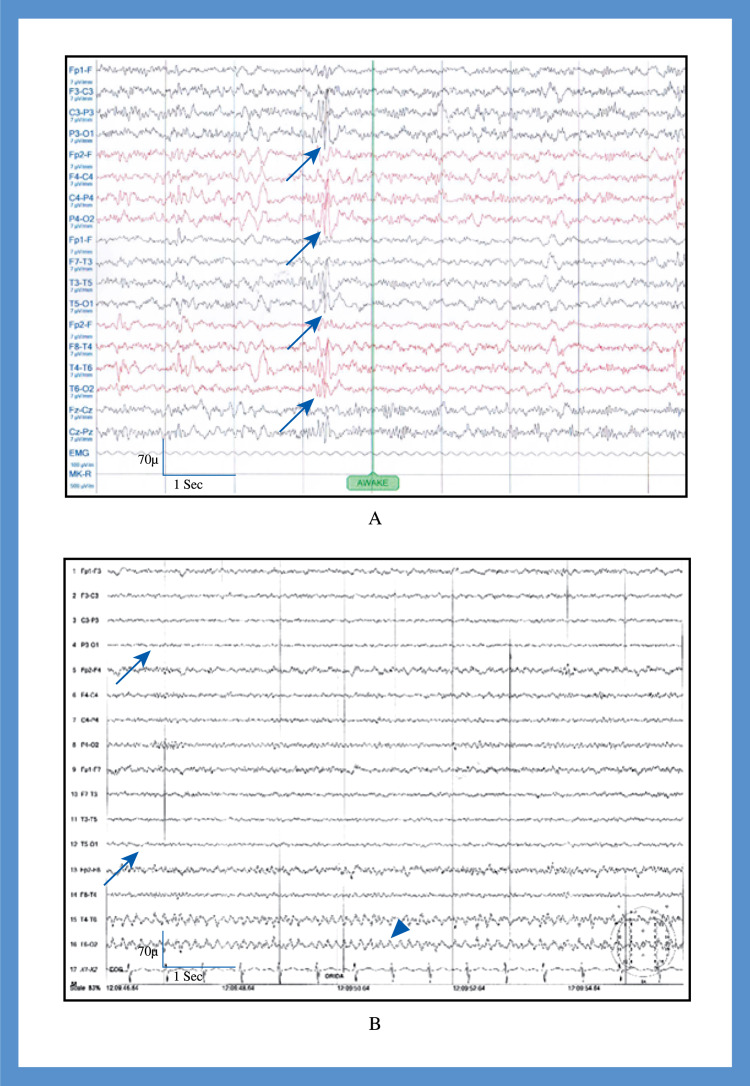
Electroencephalogram of patients with occipital epilepsy - cases 8 and 10 (A) Case 8, recorded in awake state with sensitivity 70 µV. The longitudinal bipolar montage revealed bilateral occipital epileptiform discharges (arrow) with slow waves in both occipital region (arrow head). (B) Case 10, recorded in awake state with sensitivity 70 µV. The longitudinal bipolar montage revealed loss of normal background rhythm in the left occipital cortex (arrow) and slow waves in the right occipital region (arrow head).

The visual phenomena in patients with migraine with aura included scintillating scotomas (case 11), flashes of bright white light (cases 12, 16, 17, 22, 25, 26, 27, and 28), an arc of light (case 13), flickering white light (case 14), small white dots (cases 15, 19, 20, and 21), small, bright, and golden dots (case 18), and flashes of golden light (case 24) (Table 2).

Brain MRI revealed normal findings in all patients with migraine with aura. Among the patients with occipital epilepsy, three (cases 2, 3, and 6) had normal brain MRI findings. Hence, we labeled them as idiopathic occipital epilepsy. Furthermore, there were seven cases of symptomatic occipital epilepsy. Among the symptomatic cases, one had cortical dysplasia (case 1) in the left parieto-occipital region, one had multiple ring-enhancing lesions in the right occipital region (case 5), one developed encephalomalacia in the right occipital region following an ischemic stroke (case 7), one had Wilson's disease (case 9), and one had Sturge-Weber syndrome. Two patients developed scarring in the bilateral occipital region following hypoxic insults in childhood (case 8) and eclampsia (case 4) (Table 2). The occipital cortex of the patient with Wilson's disease was hyperintense and had characteristic changes. We identified a small arachnoid cyst in the right hippocampal area in a patient (case 2) but could not determine whether this caused symptoms, so we labeled it as idiopathic (Figure 2).

The EEG findings of patients with migraine with aura were normal. It was recorded interictally. The identified EEG abnormalities in patients with occipital epilepsy were focal to bilateral occipital discharges, generalized epileptiform discharges, and focal occipital slow waves (Figures 3-5).

The occipital epilepsy patients were followed up for 12 months; one died (case 9), one was lost to follow-up (case 5), and one continued to experience seizures at the 12-month follow-up (case 8). In the rest of the cases, the seizures were controlled (Table 2).

## Discussion

In this study, all patients with occipital epilepsy presented with visual phenomena, and most were young and female. The ictal phenomenon of occipital epilepsy was transient, often multicolored, and included formed visual hallucinations. In most cases, focal seizures cause focal to bilateral seizure episodes. The postictal headaches were usually global and compressive. Nausea was rare, and patients with occipital epilepsy did not experience osmophobia. The causes of occipital epilepsy in this study were focal cortical dysplasia, hypoxic injury, calcification, and space-occupying lesions in the occipital cortex. Here, we identified clinical findings to differentiate migraine with aura from occipital epilepsy. The visual aura of migraines persisted for minutes, occurred before the headache, and included scotomas, white or golden dots, and flashing lights. In addition, the headaches were throbbing, unilateral or bilateral, and persisted for longer durations than those in occipital epilepsy. The differences between these conditions are listed in Table 3.

**Table 3:** Differentiating features of migraine with aura and occipital epilepsy

Trait	Migraine with aura	Occipital epilepsy
Visual phenomenon	Scotoma, white or golden dots, light flashes	Colorful dots or light flashes, formed hallucinations
Duration of the visual phenomenon	Minutes	Seconds
Relationship of the visual phenomenon to headache	Always before the headache	Concurrent or following the headache
Headache duration	Longer	Shorter
Headache character	Mostly throbbing and unilateral	Mostly compressive and global
Nausea	Always associated with nausea	Not associated with nausea
Osmophobia	May have osmophobia	No osmophobia
Convulsions	None	Usually associated with convulsion

This study mostly included Bangladeshi adults and symptomatic cases of occipital epilepsy. Thus, these findings may differ in different age groups, genetic compositions, and idiopathic occipital epilepsies. Occipital epilepsy is relatively uncommon, accounting for 1.2-2.6% of all epilepsy cases [2], but its prevalence in the Bangladeshi population is unknown. In this study, among the relatively young patients, 30% were idiopathic and most patients were symptomatic; this study included more symptomatic cases than that reported by Panayiotopoulos (27%) [3]. The difference could be attributed to our exclusion of children. Panayiotopoulos syndrome and idiopathic childhood occipital epilepsy of Gastaut usually manifest between 1-14 and 1-18 years, respectively [12,13]. In our study, the lowest age in the occipital epilepsy group was 14 years, with a median age of 22 years. Most of the patients in this study were women; Panayiotopoulos reported a female preponderance [14] for benign occipital epilepsy. We found that symptomatic cases were also common in female patients.

As expected, ictal episodes in occipital epilepsy manifest as visual phenomena [1,15]. The subjective manifestations of seizures originating from the occipital region may include disturbances of body image, ictal amaurosis, elementary and complex visual hallucinations, visual illusions, and oculomotor features such as adversive/oculoclonic seizures, epileptic nystagmus, eyelid flutter, and rapid blinking [16].

Occipital cortical dysplasia is a rare developmental anomaly that usually manifests during the first 20 years of life and results in refractory epilepsy [2]. Focal cortical thickening, poor delineation of the gray-white matter interface, and gyral asymmetries are the characteristic MRI findings [17]. Our first case had typical MRI findings in the left calcarine sulcus, and the patient presented with visual seizures (elementary visual hallucination in the form of photopsias followed by ictal amaurosis in the right eye). Dysplasia is usually highly epileptogenic and may have continuous or quasi-continuous rhythmic spiking, rhythmic bursts, or repetitive electrographic seizures [18]. This patient had subclinical generalized epileptiform discharges of 4-5 Hz with posterior dominance. Our initial diagnosis was migraine with aura; however, the patient’s symptoms did not improve even after two months of standard migraine treatment. Therefore, brain MRI and EEG were performed. We then administered an escalating dose of carbamazepine, after which the patient was symptom-free within six months of follow-up.

Arachnoid cysts occur in all age groups and are rarely symptomatic [19]; seizures have been reported only in a few cases [19]. Our second case was also treated for migraine with aura and showed a poor response even after three months of standard treatment. Therefore, we performed brain MRI and EEG and incidentally identified an arachnoid cyst in the right hippocampal area. Convulsions originating in the hippocampus will give rise to mesial temporal lobe epilepsy, which may be focal, focal with impaired consciousness, or focal with bilateral seizures [20]. Focal convulsions in this area mainly manifest as autonomic/psychic symptoms and auditory or olfactory phenomena that may have automatism in focal seizures with impaired consciousness [20]. The ictal semiology of this patient was consistent with an occipital rather than temporal lobe semiology, and the epileptic discharges were also observed in the occipital region. Therefore, we considered the arachnoid cyst to be an incidental finding. We then treated the patient with carbamazepine, and the patient was seizure-free after one month.

Eclampsia/preeclampsia is a systemic disease with multiorgan endotheliopathy that may cause cerebral dysfunction due to impaired cerebral blood flow autoregulation [21]. This disease may also be associated with posterior reversible encephalopathy syndrome [21,22], which shows unique neuroimaging findings in brain MRI, including bilateral white matter changes suggestive of brain edema predominantly involving the occipital region that may also affect the brainstem, cerebellum, and other watershed areas of the cerebral hemisphere [22]. Although MRI changes are reversible within three months of diagnosis [23], delayed diagnosis and treatment-induced hypotension may produce chronic neurological sequelae that may result in occipital epilepsy [23]. Our patient had a history of eclampsia at 25 years old. Brain MRI showed permanent changes in the occipital region, and she developed features suggestive of occipital epilepsy (clinical and EEG) in her fifth decade. After two months of carbamazepine treatment, the patient was seizure-free.

Our fifth patient presented with focal (visual seizure in the form of photopsias followed by ictal amaurosis) to bilateral tonic-clonic seizures. She had multiple ring-enhancing lesions in the right parieto-occipital region. Adcock and Panayiotopoulos [1] reported that a focal seizure originating from a supracalcarine lesion tended to propagate to the parietal and frontal areas. The focal seizure may also spread bilaterally, giving rise to generalized tonic-clonic convulsions. Our patient also had Todd's paresis on her right side, as well as vertigo and tinnitus, which indicated a spread in the posterior temporal lobe [2]. The patient was referred to the neurosurgery department but was lost to follow-up; thus, she was not evaluated further for the identification of the etiology of the ring-enhancing lesion.

Two or more unprovoked seizures occurring two weeks after stroke onset define post-stroke epilepsy, which may occur in 3-30% of cases [24]. This condition occurs due to hyperexcitability and synchronization of the anatomical and physiological alterations of the injured brain area [25]. Case number seven had a history of ischemic stroke with right hemianopia; encephalomalacia eventually developed in the left occipital region. He presented with focal visual seizures with a tonic deviation of the eye and ipsilateral head-turning, which are common presentations of occipital epilepsy [3]. Even after increasing the dose of carbamazepine, his epilepsy remained poorly controlled, and his focal encephalomalacia sometimes became intractable.

Neonatal hypoglycemia, hypoxia, and convulsions may cause preferential encephalomalacic changes in the parieto-occipital region due to unexplained reasons [26]. Case number nine experienced seizures at one and a half months of age. Due to the lack of documentation, we were unable to identify the reasons for these seizures. The patient presented with focal visual to bilateral tonic-clonic seizures. An MRI of the brain revealed bilateral encephalomalacia in the occipital region.

The prevalence of epilepsy is ten times higher in patients with Wilson’s disease, and approximately 6% of patients develop seizures, mainly partial seizures [27]. The causes of epilepsy in Wilson’s disease are multifactorial, among which is the direct deposition of copper in the cortex and white matter [28]. The patient with Wilson’s disease had complex hallucinations followed by generalized seizures. There was a bi-frontal slow wave with right-sided occipital epileptiform discharges. The patient’s symptoms presented at a very advanced stage, and hepatic decompensation had already developed. Levetiracetam was administered to this patient. Unfortunately, the patient died after one month.

Sporadic somatic mutations in the G protein subunit alpha Q on chromosome 9 cause Sturge-Weber syndrome [29]. Leptomeningeal angiomata (most often involving the occipital and posterior parietal lobes) lead to calcification [29], which may cause symptomatic occipital epilepsy [30]. Case number 10 had focal visual seizures that spread frontally, resulting in focal seizures in the left hand. The patient responded well to carbamazepine treatment.

Among the idiopathic cases, two patients presented with visual seizures and photopsias, whereas one patient presented with complex visual hallucinations; all responded well to treatment. This single-center cross-sectional study included a limited number of patients. As most patients with occipital epilepsy were symptomatic and adults, we could not differentiate between the presentations in different age groups and symptomatic or idiopathic causes.

## Conclusions

The diagnosis of occipital epilepsy can be missed owing to its similar presentation to migraine with aura. A meticulous history of the aura of the migraine should be taken. Patients presenting with very transient and distinct elementary or complex visual hallucinations, headaches with characteristics different from those of migraine, and associated convulsions warrant evaluation for occipital epilepsy. The appropriate anti-epileptic drug helps in quick remission of the seizure in occipital epilepsy.
